# Occurrence of viral pathogens in *Penaeus monodon* post-larvae from aquaculture hatcheries

**DOI:** 10.1016/j.dib.2015.05.009

**Published:** 2015-05-26

**Authors:** Toms C. Joseph, Roswin James, L. Anbu Rajan, P.K. Surendran, K.V. Lalitha

**Affiliations:** a Microbiology, Fermentation and Biotechnology Division, Central Institute of Fisheries Technology, Cochin 682029, Kerala, India; b Poothuvallil, Dr. Surendran lane, Perumpadappu, Palluruthy P.O, Cochin 682006, Kerala, India

**Keywords:** *Penaeus monodon* post-larvae, Polymerase Chain Reaction, Hepatopancreas Parvovirus, Infectious Hypodermal and Hematopoietic Necrosois Virus, Monodon Baculovirus, White Spot Syndrome Virus

## Abstract

Viral pathogens appear to exert the most signiﬁcant constraints on the growth and survival of crustaceans under culture conditions. The prevalence of viral pathogens White Spot Syndrome Virus (WSSV), Hepatopancreatic Parvo Virus (HPV), Monodon Baculo Virus (MBV) and Infectious Hypodermal and Hematopoietic Necrosis Virus (IHHNV) in *Penaeus monodon* post-larvae was studied. Samples collected from different hatcheries and also samples submitted by farmers from Kerala were analyzed. Out of 104 samples collected, WSSV was detected in 12.5% of the post-larvae samples. Prevalence of concurrent infections by HPV, MBV and WSSV (either dual or triple infection) was present in 60.6% of the total post-larvae tested. Out of the 51 double positives, 98% showed either HPV or IHHNV infection. HPV or IHHNV was detected in 11 post-larval samples showing triple viral infection. This is the first report of IHHNV from India. Result of this study reveals the lack of efficient screening strategies to eradicate viruses in hatchery reared post-larvae.

## 1 Introduction

Asia has always led world production of cultivated shrimp with a market value of billions of US dollars per year. India is ranked among the top five shrimp farming countries globally. It is one of the largest producers of the black tiger shrimp *Penaeus monodon* in the world. It contributed 21% and 44% by volume and value, respectively to Indian seafood exports in 2008–09. Current estimates predict that up to 40% of tropical shrimp production (>$3bn) is lost annually, mainly due to viral pathogens for which standard preventative measures (e.g. such as vaccination) are not feasible. In 1996 and 1997 WSSV was more disastrous, with cumulative lost export revenue estimated at 1 billion US dollars [7]. The white spot disease (WSD) has rapidly spread to different regions of the world with an economic impact approaching US$ 10 billion.

In shrimp culture the WSSV is one of the most devastating viruses infecting penaeid shrimp. White spot viral disease has caused high mortalities and severe damage to the shrimp culture industry in India. This virus affects all life stages of *P. monodon* and mortality rate can reach 100% within 3–9 days of the onset of clinical signs [21]. The white spot disease virus is believed to have been transmitted through seed brought to India clandestinely from Southeast Asian countries, where the virus has been amplified before. WSSV has been recorded from a wide range of wild crustaceans including crabs, lobsters and shrimp [21,20,42,30,12] as well as hatchery reared post-larvae from Asia [11,29,39].

Viral diseases due to Monodon Baculovirus (MBV) and Hepatopancreas Parvovirus (HPV) in hatchery reared larvae have been reported in India [6,36,24]. Although MBV is relatively well tolerated by *P. monodon* [5] it has been implicated in mass mortalities in shrimp cultured at high densities [10]. Infectious Hypodermal and Hematopoietic Necrosis Virus (IHHNV) is one of the major viral pathogens of penaeid shrimps worldwide [16,7] and has been found to be widely distributed in wild and cultured *P. monodon* in east and SE Asia [37].

Even though IHHNV infection does not cause mortality in *P. vannamei* and *P. monodon*; it results in a disease called Runt Deformity Syndrome in both species [3,35] and hence causes substantial economic losses [43]. In India, there has been a considerable increase in the culture of *P. monodon* due to its taste, market demand both national and international markets and because of the recent introduction of *Litopenaeus vannamei*, it has become an issue of concern and more detailed studies are essential to understand the diversity of viral pathogens in hatcheries. Viral pathogens appear to exert the most signiﬁcant constraints on the growth and survival of crustaceans under culture conditions. Expansion of the aquaculture industry, and the increasingly globalized trade in brood stock, larvae and commodity products arising from shrimp farming led to trans-boundary movements the viral pathogens and emergence of several signiﬁcant disease conditions in the country. In light of this problem, new approaches are urgently required to enhance yield by improving brood stock and larval sourcing, and promoting best management practices to control disease problems. Methods for prevention of viral infection in shrimp ponds are possibly through avoidance by screening the PL׳s before stocking shrimp in the pond. The diagnosis of viral pathogens and the rapid enforcement of biosecurity measures to avert the subsequent spread of pathogens is central to the prevention of epidemics. The objective of the present study was to determine the viral pathogens in post-larvae of *P. monodon* from hatcheries in India by Polymerase Chain Reaction to understand the viral diversity in *P. monodon* hatcheries to enforce biosecurity measures.

## 2 Materials and methods

### 2.1 Collection of samples

Samples of *P. monodon* post-larvae were randomly collected from commercial hatcheries in Kerala (both Government and private), India. Post-larvae (200 Nos.) from each hatchery were collected in polythene bags with oxygenated water and transported to the laboratory for further analysis. On arrival in the laboratory the post-larvae was frozen in liquid nitrogen and then stored at −70 °C.

### 2.2 Extraction of DNA

Viral DNA was extracted from the post-larvae samples as described by Otta et al. [29]. Total DNA extracted from infected *P. monodon* was used as positive control.

### 2.3 Polymerase Chain Reaction

For detection of IHHNV two sets of primers were used (Table 1). The PCR reactions were done as described in OIE Manual of Diagnosis for Aquatic Animals [28]. For HPV detection, two sets of primers were employed. The first set utilizes a nested PCR with primers (H441F and H441R) for first step PCR and primers (HPVnF and HPVnR) for nested PCR. The PCR protocol for the first PCR was done as described by Phromjai et al. [34] and the nested PCR as described by Umesha et al. [40]. The second set utilized the PCR protocol and primers (1120F and 1120R) as described by Pantoja and Lightner [31]. MBV was detected as described by Belcher and Young [2]. For amplification of WSSV, nested PCR were done with primers (IK1 and IK2, IK3 and IK4) as described by Umesha et al. [40]. A second set of primers was also used for the detection of WSSV using primers and PCR conditions as described by Kimura et al. [14]. The amplified PCR products were analyzed in 1.5% agarose gel containing ethidium bromide at a concentration of 0.5 µg ml^−1^ and analyzed using a gel documentation system (Alpha Imager, 1220, Alpha Innotech). A 100 bp DNA ladder was included in the gel as the molecular weight marker.

## 3 Results

Of 104 post-larvae samples tested, 4.8% samples were uninfected. All the four viruses were not detected in any samples. Three viruses HPV/MBV/IHHNV and HPV/IHHNV/WSSV were detected in 4.8% samples. Moreover, 13 (12.5%) of samples were infected only with WSSV. 79 (76%) were positive for IHHNV, 65 (62.5%) were positive for HPV and 13 (12.5%) were positive for MBV (Table 2). Dual to triple infection was present in 60.6% of the total post-larvae tested. Out of the 51 double positives 50 (98%) included either HPV or IHHNV infection. HPV or IHHNV was present in 11 (100%) post-larval samples found positive for triple viral infection. A total of 99/104 (95.2%) post-larval samples were positive for HPV and IHHNV alone or in combination with other viruses. HPV or IHHNV was present alone in 36 (34.6%) of the total post-larval samples tested. Out of the 99 samples infected with virus, 79 (79.8%) of the samples had IHHNV alone or in combination with other viruses while HPV was present in 65 (65.6%) of the samples tested.

With respect to multiple and single viral infections detected in the 95 hatchery samples by PCR (Table 2), 26 (27%) were positive for all 3 viruses (HPV, MBV and WSSV), 1 (1%) was positive for HPV and MBV, 3 (3%) were positive for HPV and WSSV, 21 (22%) were positive for MBV and WSSV, and 2 (2%) were positive for HPV only. MBV alone was detected in 8 samples (8%) and WSSV alone in 26 (27%). No viruses were detected in only 8 (8%) of the 95 samples. PCR analysis for multiple and single viral infections in 107 samples submitted by farmers (Table 3) revealed that 31 (29%) were positive for all 3 viruses (HPV, MBV and WSSV), 1 (1%) was positive for HPV and MBV, 1 (1%) was positive for HPV and WSSV, and 20 (19%) were positive for WSSV and MBV. MBV alone was detected in 10 samples (9%), and WSSV alone in 27 samples (25%), while no viruses were detected in 17 samples (16%). These results indicate not only a high prevalence of all the 3 viruses in *P. monodon* PL in India, but also a high prevalence of concurrent viral infections that usually include HPV. A significant number of samples (22% of hatchery samples and 19% of farmer samples) also showed dual infections of MBV and WSSV, as reported earlier by Otta et al. [29]. The presence of concurrent viral infections causing mortality in *P. monodon* PL has also been recorded by Manivannan et al. [24]. Thus, it is concluded that screening of PL for all these viruses is essential before stocking of culture ponds. The presence of multiple viruses may impact the health of *P. monodon* PL. The observation that infection with HPV alone occurs in only a small percentage (2%) of *P. monodon* PL may be significant. Similarly, infection with MBV alone was observed in only 8% of hatchery samples and 9% of samples submitted by farmers, while infection with WSSV alone was found in 27% of hatchery samples and 25% of samples submitted by farmers (Tables 2 and 3). The results suggest that HPV and MBV are mostly found as components of multiple viral infections, while WSSV is more frequently found alone.

## 4 Discussion

Rapid detection of viral pathogens in post-larvae would be very essential for effective health management in aquaculture. Both one step methods and nested PCR methods have been described for detection of WSSV. The nested PCR amplification procedure is 10^3^–10^4^ fold more sensitive than the one step PCR method [21,20]. The carrier state of WSSV gives only nested PCR test results [33]. Introduction of stress to shrimp by environmental factors such as pH, salinity, temperature, water level [12] may convert the pre-patent carrier state to the patent infecting state within few days or even hours [13,18,32,33] there by giving a first step PCR positive reaction. The presence of WSSV has been reported in wild broodstock from Taiwan, Japan and India [19,^30^,39]. The prevalence of WSSV in post-larvae has been found to be much lower compared to the prevalence in broodstock [19,39]. The presence of WSSV has been reported in apparently healthy post-larvae by PCR [19,^30^,^23^,^29^,39]. In the present study, WSSV was detected in 2.9% of the post-larvae tested by first step PCR and 12.5% by nested PCR. WSSV has been found to be highly prevalent among post-larvae samples from hatcheries of India. The 12.5% prevalence of WSSV in post-larvae reported in this study is comparatively lower than 75% prevalence reported by Otta et al. [29] but was similar to 12.4% prevalence reported by Uma et al. [39].

The PCR primers used for the detection of MBV gave an amplified product of 533 bp for the first reaction and a 361 bp fragment in the nested reaction. Mortalities of shrimp larvae due to MBV have been reported in many countries [1,24]. MBV is reported to be well tolerated by *P. monodon* [5,17]. The prevalence of MBV in post-larvae ranged from 25% to 92% in various hatcheries in India [36,30,39]. MBV is transmitted by oral route from water contaminated with virus from fecal matter of brood stock [4]. MBV was found to be prevalent in almost 40% of the wild shrimp seed in Vietnam by histological examination [11]. MBV has also been reported in female brood stock in Thailand with a prevalence of 33% in 1987 and 100% in 1989 [15]. The results of this study indicate a very low prevalence of MBV in post-larvae from hatcheries of India compared to 68–92% prevalence by a wet squash method [36] and 39–54% by PCR [29,39]. The low levels of infection the post-larvae in the present study may be due to improved hygiene practices in the hatchery.

HPV was reported in wild *P. monodon* and hatchery reared larvae with prevalence ranging from 31% to 62% [41,25,40]. All the post-larval samples were negative for HPV with the primers described by Pantoja and Lightner [31]. The results of the present study indicate that the strains of HPV present in the post-larval samples are similar to that of HPVmon isolated from *P. monodon* from Thailand and not HPVchin isolated from *P. monodon* in China. Umesha et al. [40] also reported the presence of only HPVmon in *P. monodon* from shrimp ponds from India. *P. monodon* infected with HPV rarely show gross signs of disease [38]. However Umesha et al. [40] has noted no difference in production in HPV infected and HPV uninfected farms. Multiple virus infection with MBV, HPV and WSSV in *P. monodon* post-larvae in India has been implicated as cause for mortality [24]. In the present study, HPV was present in 62.5% of the post-larval samples.

IHHNV is one of the highly pathogenic viruses of penaeid shrimp and has been studied since its discovery in 1983. IHHNV was found in 51.5% of penaeid shrimp culture in China [44].

The prevalence of IHHNV from wild caught *L. vannamei* broodstock captured off the Pacific coast of Panama was 20% by dot blot assay [27]. The virus can be transmitted horizontally through ingestion of infected and dead animals [3,22]. The virus can be transmitted vertically also from infected females to the embryos [26]. In the present study, IHHNV was present in 76% *P. monodon* post-larval samples. IHHNV infection is found to be well tolerated by *P. monodon* [16,8,9]. However there is a possibility of transmitting the virus to species that are susceptible.

There is very little data on the simultaneous presence of WSSV, MBV, HPV and IHHNV in *P. monodon* post-larvae meant for stocking in aquaculture ponds and there had been no studies on the prevalence of IHHNV in India. In this study, HPV and IHHNV alone or in combination was detected in 93.3% of the samples. It can be assumed that the very high rates of prevalence of HPV and IHHNV in samples are primarily due to lack of screening strategies for the presence of these viruses in India. Hence measures are yet to be initiated for control of HPV and IHHNV infection in shrimp. It is worthwhile to note that the percentage of hatchery reared post-larvae infected with WSSV and MBV is less. This is due to the stringent screening strategies initiated by hatcheries.

## Figures and Tables

**Table 1:** Primers used for the detection of shrimp viruses.

**Virus**	**Name**	**Sequence**	**Size (bp)**	**Reference**
**IHHNV**	77112F	5′ATC GGT GCA CTA CTC GGA 3′	356	
	77012R	5′TCGTACTGGCTGTTCATC3′		
	389F	5′ CGG AAC ACA ACC CGA CTT TA 3′	389	
	389R	GGC CAA GAC CAA AATACGAA3′		
**HPV**	H441F	5′GCATTACAAGAGCCAAGCAG3′	441	[34]
	H441R	5′ACA CTC AGCCTC TACCTTGT3′		
	HPVnF	5′ATA GAA CGC ATA GAA AAC GCT3′	265	[40]
	HPVnR	5′CAG CGA TTC ATT CCA GCG CCA CC 3′		
	1120F	5′GGT GAT GTG GAG GAG AGA3′	592	[31]
	1120R	5′GTA ACT ATC GCC GCC AAC3′		
**WSSV**	IK1	5′TGG CAT GAC AAC GGC AGG AG 3′	486	[40]
	IK2	5′GGC TTC TGA GAT GAG GAC GG3′		
	IK3	5′TGT CAT CGC CAG CAC GTG TGC3′	310	
	IK4	5′AGA GGT CGT CAG AGC CTA GTC3′		
	WSSV1OUT	5′ATC ATG GCT GCT TCA CAG AC 3′	982	[14]
	WSSV2OUT	5′GGC TGG AGA GGA CAAGACAT3′		
	WSSV1 in	5′TCT TCA TCA GAT GCT ACT GC3′	570	
	WSSV2 in	5′TAA GGC TAT CCA GTA TCA CG3′		
**MBV**	MBV14F	5′CGATTCCAT ATCGGC CGAATA	533	
	MBV14R	5′TTG GCATGCACTCCCTGAGAT		
	MBV14NF	5′TCCAATCGC GTCTGCGAT ACT3′	361	[2]
	MBV14NR	5′CGC TAAA TGG GGC ACA AGT CTC3′		

**Table 2:** Prevalence of multiple viral pathogens hepatopancreatic parvovirus (HPV), Monodon Baculovirus (MBV), White Spot Syndrome Virus (WSSV) and Infectious Hypodermal and Hematopoietic Necrosis Virus (IHHNV) in *Penaeus monodon* post-larvae from hatcheries in Kerala, India.

Total no. of samples analyzed	No. positive for HPV, MBV WSSV	No. positive for HPV, MBV, IHHNV	No. positive for MBV, IHHNV WSSV	No. positive for HPV, IHHNV WSSV	No. positive for HPV MBV	No. positive for HPV WSSV	No. positive for HPV IHHNV	No. positive for IHHNV WSSV	No. positive for MBV, WSSV	No. positive for MBV IHHNV
104	0	5 (4.8%)	1 (0.96%)	5 (4.8%)	1 (0.96%)	5 (4.8%)	37 (35.6%)	1 (0.96%)	1 (0.96%)	6 (5.8%)

**Table 3:** Prevalence of single viral pathogens hepatopancreatic parvovirus (HPV), Monodon Baculovirus (MBV), White Spot Syndrome Virus (WSSV) and Infectious Hypodermal and Hematopoietic Necrosis Virus (IHHNV) in *Penaeus monodon* post-larvae from hatcheries in Kerala, India.

Total no. of samples analyzed	No. of uninfected samples	No. positive for WSSV alone	No. positive for HPV alone	No. positive for MBV alone	No. positive for IHHNV alone	Total no. positive for WSSV	Total no. positive for HPV	Total no. positive for MBV	Total no. positive for IHHNV
104	5 (4.8%)	1 (0.96%)	12 (11.5%)	0	24 (23.1%)	13 (12.5%)	65 (62.5%)	13 (12.5%)	79 (76%)
